# Supplementary material for: Patterns of Intron Gain and Loss in Fungi
Source: PLoS Biol. 2004 Nov 30;2(12):e422. doi: 10.1371/journal.pbio.0020422 (PMC532390; doi:10.1371/journal.pbio.0020422)
Supplement: Table S1 — Also available at http://genes.mit.edu/NielsenEtAl/. (4.3 MB ZIP). [file pbio.0020422.st001.zip › NielsenEtAl/html/806.html]

AN5778.1.NCU02781.1.MG04352.1.FG00610.1


```
 CLUSTAL W (1.82) Multiple Sequence Alignments - Introns Inserted


Sequence 1: NCU02781.1	368 aa
Sequence 2: FG00610.1	363 aa
Sequence 3: MG04352.1	363 aa
Sequence 4: AN5778.1	363 aa
Alignment Length: 368 aa
Number Identitical Residues: 237 aa
Alignment Score (without introns) 11197


MG04352.1 	MAPPEVHHLFHNPIADHSFSADRQTLAIARDSTVELYGRAEKGFRLQDELKGHDKTVTSV
NCU02781.1	MAAPEVHHLFHHPIADHSFSADRQTLAIARDTQVELYGRVGNAFKLKDELKGHDKLVTSV
FG00610.1 	MGAPEVHHLFHNPIADHSFSADQSTLAVARDTSVELYGKVGSAFKLKDSLKGHDKTVTGV
AN5778.1  	MATAEPHHLFHAPIADHSFSSDKSVLAVARENNVELYQKTGSKFSLTDELKGHEKTVTSV
          	*...* ***** ********:*:..**:**:. **** :. . * * *.****:* **.*

MG04352.1 	DIAPSSGRIVTCSQD1RNALVWEPSPTGYKPTLVLLRINRAATYVRWSPSETKFAVGSGD
NCU02781.1	DIAPNTGRIVTCSQD1RNALVWEPSPTGYKPTLVLLRISRAATFVRWSPSEAKFAVGSGD
FG00610.1 	DIAPNSGRIVTCSQD1RNAIVWEPSPEGYKPTLVLLRISRAATFVRWSPSENKFAVGSGD
AN5778.1  	DIAPNSGRIVTCSQD1RNAYVWEQTPTGWKPTLVLLRINRAATFVRWSPSEQKFAVGSGA
          	****.:********* *** *** :* *:*********.****:******* ******* 

MG04352.1 	RVIAICYFEEENDWWVSKHLKKPIRSTITTVAWHPNSVLLAAGSTDAHARVLSSFIKGVD
NCU02781.1	RVIAICYFEEENDWWVSKHLKKPIRSTITSVAWHPNSVLLAAGSTDAHARVFSAFIKGTD
FG00610.1 	RVIAVCYFEEENDWWVSKHLKKPIRSTITSVAWHPNSVLLAAGSTDAHARVFSAFIKGMD
AN5778.1  	RVIAVCYFEEENDWWISKHLKKPIRSTITTLAWHPNSVLLAAGSTDSHARVFSSFIKGVD
          	****:**********:*************::***************:****:*:**** *

MG04352.1 	TKPEPTGWGERLPFNTVCGEYLNNSAGWVHAVAFSPSGEALAFAAHDSSITVVYPGGPDQ
NCU02781.1	QRPEPSPWGERLPFNTVCGEFLNNSAGWVHSVAFSPSGDALAFAAHDSSITVVYPSAPEQ
FG00610.1 	ARPPPGVWGERLPFNTVCGEYLNNSAGWVHSVSFSPSGDSLAFAAHDSSITVVYPSGPDQ
AN5778.1  	TRPEPSAWGERLPFNTICGEFLNDSAGWIHGVCFSPSGNALAFTGHDSSVTIVYPSAPEQ
          	 :* *  *********:***:**:****:*.*.*****::***:.****:*:***..*:*

MG04352.1 	PPRALISINTQLLPFMGLVWNGESEIIAAGY~DCEAFRFRGGEGGWQLSGSLESK---GR
NCU02781.1	PPKAVISINTQLLPFMSLFWNGETEIIAAGY~DCEAFRFQGGEQGWQLAGTLEKAKAGGR
FG00610.1 	APRAVVTVSTQQLPFKSLIWTSEDEIIAAGY~DCEAFRFQGGEGGWQLAGPIEQK---GG
AN5778.1  	PPRAMLNISTRLLPFNSLIWNGENEIIAAGH0DCEPYRLRGDENGWQLEGTIENK---AG
          	.*:*::.:.*: *** .*.*..* ******: ***.:*::*.* **** *.:*.    . 

MG04352.1 	PGAD-AEREESALNMFRQMDLKGKV-KDDTQLKTVHQNTITTIRSFQESGSGLVKMSS1S
NCU02781.1	PGLGDEDREESALNMFKQMDLKGKVSKDDTMLKTVHQNTVTMLRPFEESGGRVTKFSS1S
FG00610.1 	PGLG-EHREESALNMFRQMDLKGKA-KDDTQLQTTHQNTISTVRPFESSGDRVTKFST1S
AN5778.1  	AGAG-SVREESALNMFRQMDLKGQT-QADTQLKTVHQNTINTIRVYEDTAGSVHKIST1S
          	.* .   *********:******:. : ** *:*.****:. :* ::.:.. : *:*: *

MG04352.1 	GVDGRIVIWDV
NCU02781.1	GVDGRVVIWHT
FG00610.1 	GVDGRIVLWSA
AN5778.1  	GVDGRVVIWTI
          	*****:*:*
```
